# Supplementary material for: Multi-GPU MBE(3)-OSV-MP2 for Performant Large-Scale Ab Initio Calculations
Source: J Chem Theory Comput. 2026 Jul 8;22(14):7249–63. doi: 10.1021/acs.jctc.6c01147 (PMC13420557; doi:10.1021/acs.jctc.6c01147)
Supplement: Supplementary file 1 [file ct6c01147_si_001.pdf]

Supporting Information

Multi-GPU MBE(3)-OSV-MP2 for Performant Large-Scale *ab initio* Calculations

Qiujiang Liang[†,‡] and Jun Yang[*,†,‡,¶]

†*Department of Chemistry, The University of Hong Kong, Hong Kong 999077, P.R. China*

‡*Hong Kong Quantum AI Lab Limited, Hong Kong 999077, P.R. China*

¶*HKU-CAS Joint Laboratory on New Materials, The University of Hong Kong, Hong Kong 999077, P.R. China*

E-mail: juny@hku.hk

S1 Results

Table S1: Percentage of canonical RI-MP2 correlation energy recovered by GPU-based and CPU-based[1] MBE(3)-OSV-MP2 for various molecular systems.

			$E_c^{\text{MBE(3)-OSV-MP2}}/E_c^{\text{RI-MP2}}$	
	basis	N_{ao}	CPU[1] (%)	GPU (%)
DIAD	def2-TZVP	1392	99.67	99.68
BHS	def2-TZVP	1586	99.80	99.81
Nonactin	def2-TZVP	1996	99.81	99.86
YIVNOG	def2-TZVP	2046	99.86	99.87
FLP	def2-TZVP	2059	99.69	99.71
$(H_2O)_{32}$	aug-cc-pVTZ	2944	99.92	99.97

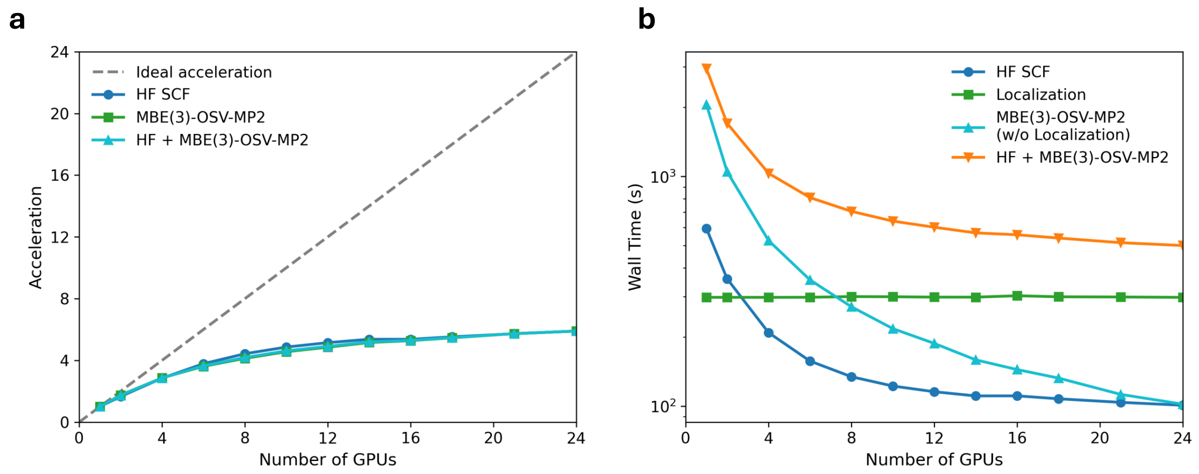

Fig. S1: Parallel performance of the GPU based HF SCF and MBE(3)-OSV-MP2 calculations. (a) Acceleration relative to a single GPU as a function of the number of GPUs. (b) Wall time for key steps as a function of the number of GPUs. Calculations were performed using a $(H_2O)_{300}$ cluster with cc-pVDZ/cc-pVDZ-RIFit basis sets.

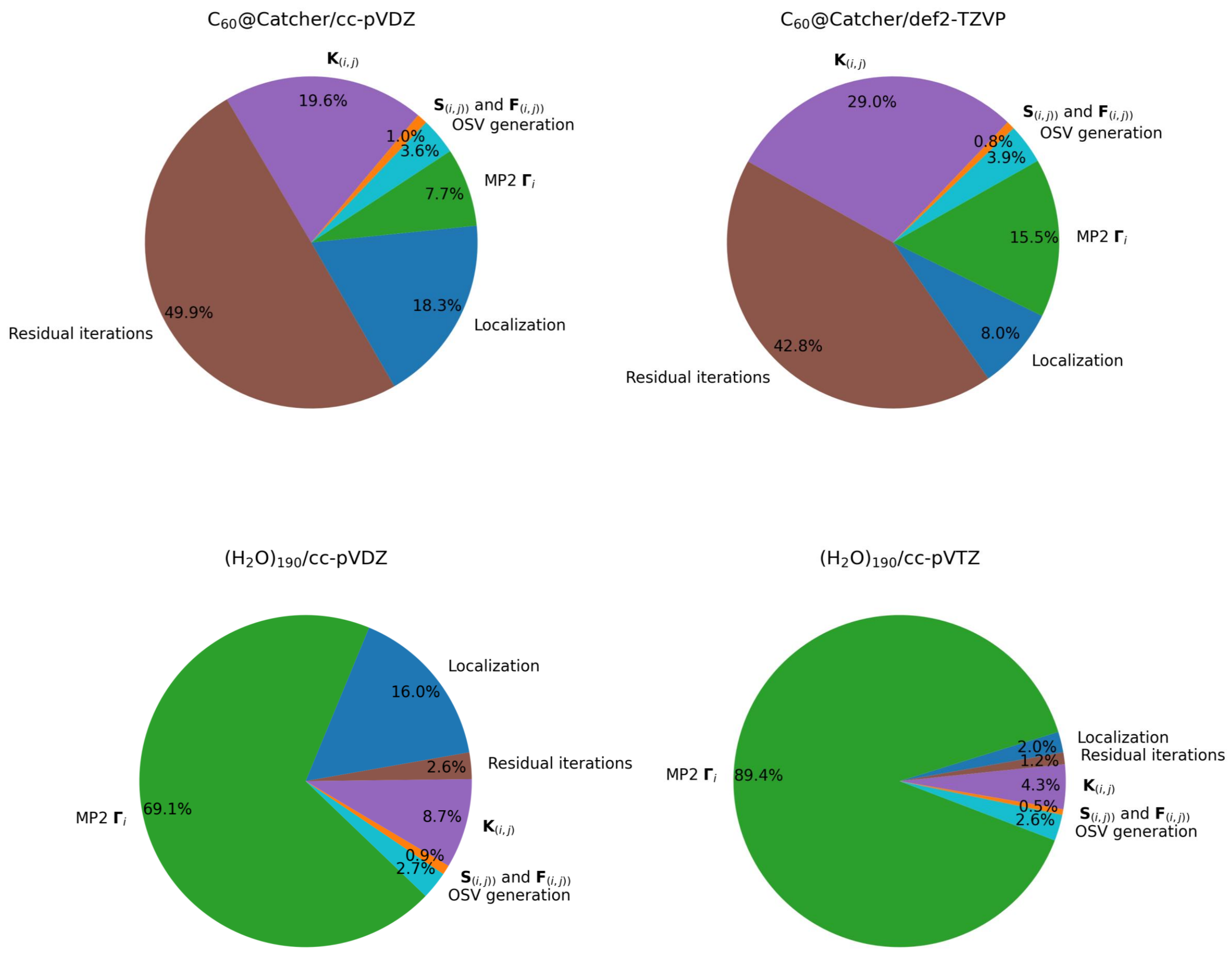

Fig. S2: Percentage breakdown of total wall time for the GPU-accelerated MBE(3)-OSV-MP2 calculations on C_{60}@Catcher and $(H_2O)_{190}$ with various basis sets.

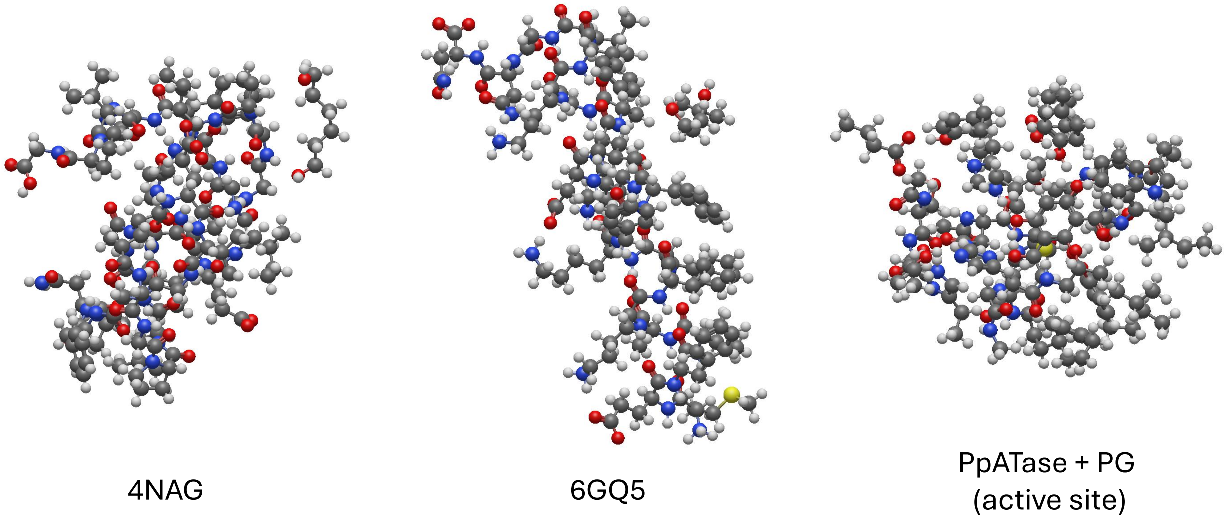

Fig. S3: Protein structures simulated in this work. The structures of 4NAG and 6GQ5 were obtained from Ref. 2, while the active site configuration of PpATase + PG was sourced from Ref. 3.

S2 Algorithms

Table S2: Custom Kernels developed in this work

Steps	Kernels
Γ_i generation	sparseHalfTransKernel
Localization	pijaLowdinKernel<br>computeRotAnglesKernel<br>reduceAijBij1BlockKernel<br>rotateUKernel<br>rotatePijaRowKernel
OSV generation	normalizeVec1BlockKernel<br>batchedDnorm2Kernel<br>findMaxValue1BlockKernel
OSV S/F	osvSFKernel
OSV K	imujpKernel<br>closeOsvKmatKernel<br>remoteOsvKmatKernel
OSV Residual	closeOsvPreconKernel<br>remoteOsvPreconKernel<br>closeResidualKernel<br>remoteResidualKernel<br>clusterResidualKernel

S3 Coordinates

120			
w40 optmized with ChargeNN			
O	6.195	6.913	7.494
H	5.771	6.046	7.335
H	5.530	7.589	7.269
O	4.614	9.046	7.007
H	4.747	9.412	6.114
H	3.673	8.795	7.077
O	5.085	7.063	3.937
H	5.162	7.986	4.238
H	4.826	7.124	2.992
O	4.857	4.626	7.051
H	3.894	4.527	6.904
H	5.258	4.390	6.191
O	2.445	6.683	4.992
H	3.365	6.791	4.700
H	2.297	7.287	5.740
O	5.815	4.413	4.563
H	6.782	4.344	4.449
H	5.557	5.300	4.260
O	5.274	9.741	4.467
H	5.219	9.957	3.522
H	6.128	10.112	4.765
O	8.429	8.072	6.425
H	8.768	8.482	7.246
H	7.613	7.604	6.690
O	2.134	8.074	7.371
H	2.342	7.454	8.091
H	1.734	8.836	7.827
O	8.404	4.060	3.736
H	8.375	4.943	3.332
H	7.949	3.470	3.104
O	7.681	6.576	2.900
H	8.360	7.219	3.158
H	6.840	6.872	3.284
O	9.613	8.200	3.931
H	10.542	7.959	3.907
H	9.324	8.128	4.857
O	2.800	6.772	9.709
H	2.986	7.612	10.163
H	3.399	6.118	10.113
O	7.095	7.596	9.943
H	6.689	7.198	9.147
H	6.519	7.390	10.699
O	4.351	4.945	11.063
H	4.899	4.400	10.467
H	3.563	4.377	11.170
O	5.820	9.974	9.279
H	6.355	9.203	9.535
H	5.389	9.750	8.433
O	8.142	11.043	8.080
H	7.316	10.902	8.572
H	8.802	10.454	8.477
O	5.132	3.107	9.248
H	5.088	3.640	8.430
H	5.075	2.178	8.942
O	7.645	10.542	5.463
H	7.975	9.665	5.720
H	7.769	11.077	6.266
O	2.230	4.389	6.534
H	1.650	4.543	7.296
H	2.138	5.145	5.930
O	4.609	7.556	1.372
H	3.669	7.558	1.101
H	4.925	8.472	1.307
O	9.883	4.022	6.085
H	9.447	4.025	5.216
H	9.607	3.199	6.522
O	6.467	3.013	2.139
H	5.892	3.301	2.862
H	6.393	3.691	1.442
O	6.330	5.392	0.732
H	5.583	6.008	0.742
H	7.009	5.801	1.286
O	0.892	4.958	8.897
H	0.000	5.101	9.215
H	1.442	5.688	9.232
O	7.922	2.844	9.517
H	6.955	2.926	9.540
H	8.292	3.707	9.781
O	8.151	10.315	2.788
H	8.757	9.568	2.894
H	8.043	10.675	3.684
O	6.726	0.916	6.050
H	6.653	1.319	5.182
H	7.569	1.212	6.435
O	10.149	6.209	7.626
H	9.563	6.690	7.025
H	10.264	5.340	7.195
O	3.741	9.110	10.844
H	4.221	8.566	11.487
H	4.435	9.568	10.331
O	4.925	0.732	8.066
H	4.330	0.000	7.906
H	5.571	0.738	7.332
O	2.484	3.168	10.380
H	1.875	3.583	9.751
H	3.249	2.844	9.878
O	5.655	10.128	1.776
H	6.611	10.171	1.979
H	5.452	10.919	1.273
O	1.565	9.950	9.288
H	1.157	10.780	9.536
H	2.264	9.767	9.938
O	8.870	5.391	9.938
H	9.437	5.576	9.169
H	8.269	6.141	10.032
O	9.435	8.665	8.892
H	10.045	7.931	8.717
H	8.752	8.310	9.485
O	0.884	6.106	2.806
H	0.686	5.171	2.863
H	1.377	6.340	3.612
O	1.984	7.541	0.779
H	1.537	7.072	1.510
H	1.440	7.424	0.000
O	9.001	1.735	7.358
H	9.669	1.119	7.661
H	8.608	2.132	8.165
O	5.370	7.186	12.056
H	4.996	6.320	11.794
H	5.699	7.079	12.951
240			
w80 optmized with ChargeNN			
O	7.663	6.995	9.251
H	7.393	6.106	8.952
H	6.921	7.599	9.062
O	5.949	9.046	8.814
H	6.170	9.394	7.929
H	5.066	8.633	8.742
O	6.140	7.515	5.431
H	6.398	8.352	5.863
H	5.449	7.780	4.789
O	6.881	4.512	8.460
H	6.120	4.825	7.937
H	7.526	4.176	7.810
O	4.807	5.788	7.169
H	5.230	6.244	6.423
H	4.397	6.488	7.710
O	7.690	5.331	5.796
H	8.617	5.595	5.934
H	7.186	6.149	5.644
O	6.805	9.918	6.416
H	6.456	10.787	6.141
H	7.766	10.054	6.502
O	9.688	8.672	8.838
H	9.468	9.390	9.464
H	8.971	8.013	8.914
O	3.683	7.674	8.753
H	3.787	7.328	9.660
H	2.841	8.157	8.718
O	10.300	6.102	6.077
H	10.215	7.014	5.745
H	10.765	5.618	5.368
O	7.925	7.539	3.271
H	8.743	7.875	3.673
H	7.246	7.618	3.963
O	9.950	8.502	4.856
H	10.772	8.767	4.402
H	9.765	9.180	5.526
O	4.343	6.584	11.158
H	3.733	6.952	11.825

H	5.101	6.206	11.642
O	8.190	7.343	11.868
H	8.030	7.323	10.907
H	7.538	6.722	12.238
O	6.416	5.337	12.439
H	6.692	4.574	11.896
H	6.695	5.138	13.349
O	6.449	10.667	10.880
H	6.266	10.331	11.777
H	6.161	9.971	10.259
O	9.153	10.736	10.439
H	8.223	10.676	10.714
H	9.303	11.691	10.277
O	7.233	3.285	10.820
H	7.095	3.658	9.924
H	7.366	2.322	10.698
O	9.457	10.357	6.821
H	9.643	9.709	7.528
H	9.732	11.213	7.203
O	2.814	3.995	7.673
H	2.870	4.009	8.646
H	3.602	4.475	7.359
O	4.569	8.555	3.627
H	3.603	8.706	3.648
H	4.954	9.374	3.251
O	12.111	5.603	8.052
H	11.405	5.812	7.410
H	11.978	4.675	8.297
O	6.915	3.454	4.033
H	7.059	4.102	4.751
H	6.948	3.975	3.210
O	7.045	5.283	2.015
H	6.180	5.633	1.732
H	7.493	6.047	2.426
O	2.950	4.398	10.357
H	2.103	4.643	10.762
H	3.551	5.136	10.566
O	9.990	3.538	10.925
H	9.019	3.455	10.881
H	10.190	4.460	11.177
O	10.506	11.838	4.780
H	11.107	11.321	4.223
H	10.078	11.198	5.366
O	8.923	2.912	7.364
H	9.113	2.922	6.408
H	9.776	2.917	7.822
O	11.814	7.520	10.008
H	11.071	7.887	9.495
H	12.214	6.868	9.413
O	6.178	12.550	8.745
H	6.429	13.343	9.248
H	6.318	11.801	9.346
O	7.007	0.958	7.697
H	6.738	0.730	6.786
H	7.631	1.695	7.586
O	4.572	2.274	11.101
H	3.945	2.979	10.868
H	5.462	2.654	11.083
O	7.746	11.934	3.841
H	8.689	12.027	4.036
H	7.257	12.312	4.591
O	3.448	12.374	8.279
H	3.274	12.542	7.340
H	4.411	12.417	8.390
O	10.549	5.998	11.889
H	11.167	6.501	11.331
H	9.741	6.539	11.939
O	9.841	9.070	13.292
H	10.532	9.469	12.736
H	9.326	8.475	12.721
O	1.921	8.928	3.434
H	1.824	8.182	2.818
H	1.597	8.617	4.301
O	3.161	11.430	3.390
H	2.620	10.625	3.317
H	4.006	11.256	2.936
O	11.400	2.956	8.706
H	11.904	2.179	8.959
H	10.895	3.190	9.510
O	6.436	7.299	15.448
H	6.881	6.458	15.258
H	7.093	8.009	15.353
O	1.267	8.991	8.534
H	0.747	9.776	8.340
H	0.640	8.273	8.755
O	11.643	9.999	11.288
H	10.767	10.286	10.973
H	11.822	9.123	10.902
O	11.585	4.789	4.097
H	11.600	4.566	3.144
H	12.069	4.084	4.567
O	6.748	8.678	0.972
H	6.036	8.025	0.895
H	7.351	8.333	1.648
O	6.313	0.897	5.071
H	5.358	0.781	4.931
H	6.560	1.728	4.636
O	5.825	10.329	13.507
H	6.606	10.116	14.047
H	5.133	9.707	13.789
O	3.419	12.942	5.558
H	3.103	13.794	5.254
H	3.281	12.327	4.807
O	12.712	7.374	4.150
H	12.360	6.491	3.957
H	13.256	7.269	4.952
O	4.493	0.587	8.878
H	5.362	0.718	8.464
H	4.476	1.108	9.700
O	0.737	5.263	6.641
H	1.497	4.767	7.011
H	0.058	4.613	6.446
O	4.134	8.437	14.664
H	3.764	8.769	15.484
H	4.920	7.913	14.917
O	6.089	12.499	6.006
H	6.188	12.661	6.960
H	5.151	12.657	5.796
O	2.568	1.282	7.118
H	3.173	0.890	7.781
H	2.578	2.237	7.291
O	5.675	10.712	2.482
H	6.410	11.187	2.915
H	6.083	10.166	1.783
O	1.902	4.994	3.957
H	2.685	4.405	3.974
H	1.633	5.138	4.873
O	9.554	2.842	4.677
H	8.634	2.877	4.367
H	10.015	3.587	4.260
O	2.105	9.673	11.137
H	2.322	10.626	11.185
H	1.845	9.503	10.221
O	7.478	0.664	10.338
H	6.885	0.000	10.696
H	7.362	0.646	9.368
O	7.549	4.804	14.863
H	8.443	4.579	14.538
H	7.292	4.086	15.444
O	9.297	13.349	9.930
H	9.691	13.375	9.041
H	8.529	13.936	9.955
O	4.778	6.756	1.588
H	3.848	6.578	1.392
H	4.744	7.321	2.382
O	11.914	9.819	3.429
H	12.416	9.012	3.658
H	11.837	9.826	2.473
O	7.955	9.587	15.081
H	8.377	10.079	15.786
H	8.663	9.384	14.434
O	0.000	6.662	9.002
H	0.268	6.221	9.825
H	0.225	6.082	8.257
O	10.634	12.685	7.566
H	10.831	12.768	6.621
H	11.440	12.365	8.014
O	4.069	3.381	4.015
H	3.857	2.456	4.233
H	5.035	3.446	4.005
O	2.375	7.579	12.741
H	2.219	8.395	12.218
H	2.926	7.853	13.494
O	14.054	6.757	6.461
H	13.411	6.360	7.073
H	14.927	6.503	6.762
O	1.994	6.627	1.859
H	1.326	6.345	1.235
H	1.956	5.997	2.612
O	12.045	2.712	5.890
H	11.203	2.421	5.503

H	11.892	2.840	6.834
O	5.370	12.864	12.059
H	5.293	12.587	12.977
H	5.738	12.081	11.611
O	11.424	4.143	1.508
H	10.506	4.064	1.182
H	11.994	4.024	0.748
O	1.292	7.910	5.905
H	1.422	8.328	6.768
H	1.072	6.981	6.077
O	8.890	3.895	0.575
H	8.238	4.425	1.068
H	8.393	3.313	0.000
O	12.620	11.571	9.134
H	13.541	11.779	9.295
H	12.308	11.072	9.909
O	3.581	0.780	4.726
H	3.126	0.026	4.352
H	3.188	0.929	5.613
O	9.882	4.049	13.727
H	10.299	4.858	13.387
H	9.883	3.456	12.961
O	0.815	5.707	11.500
H	1.317	6.407	11.960
H	0.164	5.385	12.125
O	2.795	12.273	10.950
H	3.617	12.576	11.361
H	2.908	12.376	9.987
O	6.774	14.593	10.456
H	6.300	14.097	11.150
H	6.441	15.490	10.481

300

w100 optmized with ChargeNN

O	11.836	9.813	15.357
H	11.611	10.408	16.072
H	11.512	10.217	14.534
O	4.065	14.160	11.264
H	4.744	13.625	11.707
H	4.422	14.411	10.397
O	8.556	12.358	13.669
H	8.065	11.619	14.074
H	9.390	11.945	13.387
O	3.183	12.678	3.389
H	2.920	11.763	3.574
H	4.089	12.639	3.036
O	2.154	10.686	11.857
H	1.828	11.598	11.838
H	2.201	10.465	10.912
O	11.316	12.876	4.352
H	12.268	12.932	4.523
H	11.054	11.993	4.668
O	11.115	6.059	0.895
H	10.482	6.697	1.280
H	10.765	5.856	0.026
O	5.691	12.063	8.066
H	6.186	11.851	7.252
H	5.130	11.288	8.255
O	10.808	7.301	15.050
H	11.120	7.106	14.148
H	11.297	8.102	15.305
O	9.519	12.152	6.996
H	9.739	11.916	7.917
H	9.844	13.061	6.854
O	4.289	11.053	13.546
H	3.488	10.931	13.009
H	4.570	10.181	13.865
O	13.912	5.226	12.569
H	13.089	5.712	12.755
H	14.498	5.864	12.133
O	9.195	9.587	3.615
H	9.905	9.815	4.241
H	8.415	9.361	4.150
O	8.531	9.196	9.478
H	8.024	8.476	9.064
H	7.923	9.946	9.620
O	8.032	6.612	5.705
H	8.926	6.948	5.908
H	7.561	7.343	5.273
O	8.968	8.933	12.134
H	8.870	9.150	11.189
H	8.463	8.106	12.233
O	7.197	7.101	8.226
H	6.318	7.391	7.922
H	7.669	6.883	7.400
O	7.273	15.650	10.291
H	7.626	15.258	11.107
H	7.054	16.561	10.501
O	4.937	8.277	11.081
H	4.296	8.322	11.816
H	5.764	7.938	11.468
O	0.170	8.618	9.355
H	0.447	7.958	10.016
H	0.187	8.168	8.496
O	5.093	9.689	2.892
H	4.166	9.652	3.185
H	5.302	10.644	2.829
O	13.934	12.508	5.297
H	13.906	11.534	5.370
H	14.709	12.723	4.778
O	5.091	4.287	11.010
H	4.447	4.896	10.603
H	5.953	4.529	10.631
O	5.002	3.534	4.361
H	5.096	2.747	3.825
H	5.897	3.801	4.631
O	11.846	4.638	9.097
H	12.350	3.975	9.598
H	11.100	4.851	9.686
O	15.114	7.155	10.963
H	14.341	7.750	10.914
H	15.880	7.712	11.108
O	6.107	12.587	12.335
H	6.892	12.614	12.901
H	5.454	12.030	12.811
O	9.036	13.746	2.962
H	9.890	13.542	3.374
H	8.841	13.016	2.355
O	6.781	8.987	4.904
H	6.898	9.864	5.327
H	6.175	9.133	4.152
O	7.608	1.954	13.632
H	6.795	2.463	13.476
H	7.508	1.549	14.496
O	11.962	11.239	10.573
H	11.102	11.650	10.348
H	12.601	11.963	10.459
O	12.433	4.459	6.367
H	12.027	3.727	5.870
H	12.057	4.424	7.263
O	12.087	7.317	8.618
H	11.402	7.744	8.063
H	11.844	6.379	8.678
O	7.497	5.180	10.054
H	7.386	5.844	9.343
H	7.610	4.318	9.600
O	2.781	14.645	5.239
H	2.235	15.284	4.778
H	2.915	13.900	4.623
O	14.842	5.765	6.321
H	14.010	5.266	6.236
H	15.169	5.520	7.200
O	2.465	14.347	8.007
H	2.444	14.384	7.039
H	3.377	14.553	8.267
O	13.560	3.291	10.772
H	13.692	3.880	11.546
H	14.187	3.633	10.112
O	11.031	10.316	5.497
H	11.988	10.135	5.540
H	10.758	10.679	6.364
O	13.268	13.601	10.229
H	12.514	14.148	10.531
H	14.052	14.005	10.602
O	4.502	9.778	8.833
H	4.705	9.311	9.664
H	3.564	10.046	8.897
O	14.665	5.205	9.055
H	14.938	5.897	9.683
H	13.706	5.299	8.944
O	0.626	7.388	6.897
H	1.472	6.909	7.004
H	0.000	6.763	6.530
O	3.046	6.251	7.211
H	3.203	6.136	8.168
H	3.753	6.853	6.916
O	7.147	6.947	12.099
H	7.264	6.248	11.432
H	6.884	6.514	12.932
O	12.838	8.637	10.817
H	12.588	9.575	10.797
H	12.509	8.254	9.979

O	9.576	14.923	8.932
H	9.818	15.002	7.990
H	8.684	15.280	9.061
O	9.721	12.324	9.754
H	8.809	12.047	9.972
H	9.695	13.289	9.606
O	4.437	1.875	9.733
H	4.873	1.979	8.874
H	4.691	2.642	10.268
O	3.610	6.238	9.856
H	2.712	6.334	10.233
H	4.110	7.008	10.182
O	9.085	0.294	11.952
H	8.486	0.838	12.484
H	9.910	0.783	11.833
O	11.424	2.497	4.714
H	11.876	1.663	4.576
H	11.529	3.003	3.886
O	13.699	6.019	1.826
H	12.804	6.237	1.508
H	14.242	5.890	1.048
O	8.554	8.364	16.149
H	8.119	7.662	16.636
H	9.379	7.985	15.792
O	13.734	9.819	5.407
H	13.932	9.129	4.750
H	13.996	9.410	6.256
O	7.200	11.398	5.923
H	7.012	12.196	5.386
H	8.081	11.573	6.312
O	11.995	3.914	2.473
H	12.795	4.460	2.481
H	11.349	4.470	2.013
O	5.162	8.426	14.207
H	5.428	7.514	14.401
H	5.984	8.947	14.275
O	5.280	3.426	13.508
H	5.199	3.708	12.573
H	4.399	3.148	13.769
O	6.753	9.045	0.792
H	6.219	8.976	0.000
H	6.135	9.139	1.540
O	5.309	15.428	6.039
H	5.300	15.252	6.993
H	4.413	15.224	5.720
O	11.111	14.977	11.137
H	10.658	15.118	10.286
H	10.405	14.925	11.795
O	0.879	4.051	11.001
H	0.088	3.539	11.173
H	1.311	3.624	10.232
O	8.445	0.178	9.239
H	7.962	1.013	9.139
H	8.510	0.000	10.191
O	14.350	7.440	4.133
H	14.721	6.834	4.800
H	14.272	6.946	3.301

O	6.882	13.692	4.638
H	7.661	14.010	4.140
H	6.442	14.458	5.054
O	10.615	14.595	6.450
H	10.681	14.239	5.550
H	11.531	14.583	6.792
O	8.235	11.287	1.692
H	7.833	10.630	1.107
H	8.693	10.758	2.371
O	1.250	6.727	11.028
H	1.623	7.088	11.851
H	1.038	5.783	11.167
O	9.681	2.760	6.814
H	10.170	2.612	5.991
H	10.148	2.219	7.482
O	10.853	1.247	8.758
H	11.137	1.437	9.660
H	10.038	0.710	8.846
O	8.675	14.871	12.628
H	8.610	13.949	12.943
H	8.649	15.416	13.417
O	5.479	2.800	7.279
H	4.570	3.049	7.028
H	6.024	3.111	6.536
O	7.492	9.919	14.236
H	7.875	9.478	15.021
H	7.966	9.550	13.469
O	1.985	2.794	8.880
H	2.773	2.331	9.223
H	2.202	3.058	7.973
O	11.797	6.581	4.737
H	12.648	6.969	4.472
H	12.002	5.775	5.243
O	5.832	12.251	2.670
H	6.147	12.814	3.404
H	6.634	12.060	2.156
O	2.784	8.193	12.849
H	2.451	9.092	12.670
H	3.453	8.263	13.551
O	1.757	9.852	6.368
H	1.893	10.272	7.232
H	1.297	9.014	6.550
O	5.045	8.040	6.856
H	5.555	8.411	6.117
H	4.813	8.777	7.456
O	11.614	1.595	11.566
H	12.107	0.808	11.815
H	12.295	2.235	11.285
O	13.098	14.119	7.444
H	13.223	13.805	8.352
H	13.502	13.485	6.832
O	7.697	2.892	8.750
H	6.857	2.787	8.260
H	8.400	2.890	8.074
O	1.968	10.568	9.081
H	1.724	11.482	9.331
H	1.183	9.994	9.215

O	1.781	12.991	10.291
H	2.504	13.426	10.782
H	1.753	13.489	9.453
O	10.858	9.983	8.297
H	11.337	10.472	8.983
H	10.021	9.709	8.720
O	7.418	3.932	5.608
H	7.612	4.888	5.630
H	8.227	3.490	5.916
O	11.675	6.813	12.519
H	12.024	7.540	11.974
H	11.046	6.322	11.963
O	9.187	7.813	1.532
H	8.341	8.111	1.167
H	9.340	8.368	2.317
O	5.206	14.622	8.726
H	5.980	14.987	9.180
H	5.421	13.695	8.499
O	10.728	10.793	13.017
H	11.271	10.997	12.239
H	10.154	10.048	12.749
O	9.880	3.329	12.984
H	10.552	2.721	12.641
H	9.079	2.799	13.142
O	14.386	8.261	7.488
H	13.576	8.023	7.970
H	14.686	7.443	7.058
O	8.947	5.261	14.872
H	9.346	4.589	14.298
H	9.627	5.946	15.007
O	3.077	3.680	6.364
H	3.546	3.636	5.515
H	3.019	4.626	6.595
O	10.035	5.192	11.018
H	9.131	5.299	10.675
H	9.969	4.526	11.729
O	10.302	7.777	6.549
H	10.475	8.735	6.511
H	10.848	7.372	5.841
O	7.220	11.465	10.172
H	6.797	11.816	10.980
H	6.621	11.697	9.437
O	2.549	10.011	3.821
H	1.709	9.849	3.389
H	2.359	9.985	4.782
O	6.336	5.841	14.436
H	7.249	5.639	14.724
H	5.928	4.989	14.210

384

w128 optmized with ChargeNN

O	9.711	10.825	11.117
H	9.372	10.049	11.597
H	8.953	11.401	10.908
O	7.893	12.696	10.323
H	8.065	12.953	9.399
H	6.973	12.363	10.371
O	8.123	10.640	7.273

H	8.370	11.504	7.657
H	7.558	10.872	6.509
O	7.235	7.400	9.871
H	6.739	8.211	9.670
H	7.823	7.299	9.101
O	6.033	9.626	8.798
H	6.712	9.949	8.186
H	5.876	10.350	9.440
O	8.848	7.954	7.743
H	9.819	7.862	7.808
H	8.665	8.903	7.658
O	8.744	13.222	7.805
H	8.452	13.743	7.035
H	9.710	13.364	7.831
O	11.704	11.815	9.557
H	12.034	12.510	10.156
H	10.945	11.420	10.026
O	5.548	11.484	10.620
H	5.780	10.852	11.330
H	4.584	11.612	10.670
O	11.580	7.979	7.659
H	11.805	8.848	7.279
H	11.827	7.314	6.990
O	10.286	10.297	5.640
H	11.136	10.382	6.110
H	9.594	10.372	6.321
O	12.365	10.507	7.297
H	13.170	10.946	6.964
H	12.132	10.986	8.117
O	6.451	9.615	12.307
H	5.968	9.509	13.147
H	7.375	9.373	12.491
O	10.739	11.608	13.537
H	10.390	11.329	12.673
H	10.700	10.825	14.123
O	9.005	8.835	12.880
H	9.025	7.957	12.450
H	8.963	8.663	13.836
O	8.661	14.481	12.104
H	8.167	14.253	12.912
H	8.399	13.796	11.456
O	11.160	14.450	10.701
H	10.303	14.491	11.153
H	11.426	15.367	10.503
O	8.933	6.410	11.706
H	8.365	6.653	10.951
H	8.691	5.491	11.940
O	11.415	13.635	7.505
H	11.640	13.030	8.234
H	11.849	14.484	7.715
O	3.906	8.054	9.312
H	3.765	8.140	10.273
H	4.667	8.621	9.086
O	6.749	11.629	5.198
H	5.904	11.223	4.950
H	6.494	12.563	5.330
O	13.764	8.586	9.298
H	13.040	8.675	8.648
H	13.716	7.662	9.598
O	7.925	6.915	5.397
H	8.142	7.246	6.287
H	8.451	7.462	4.789
O	9.129	8.702	3.745
H	8.646	9.263	3.112
H	9.610	9.297	4.349
O	3.374	8.007	12.047
H	2.435	7.813	11.885
H	3.394	8.863	12.513
O	11.531	6.361	12.193
H	10.597	6.437	11.915
H	11.510	5.789	12.983
O	12.444	13.473	4.904
H	13.027	12.703	4.787
H	12.068	13.396	5.800
O	11.016	5.011	8.761
H	11.210	5.321	7.857
H	11.718	5.366	9.324
O	13.632	10.253	11.431
H	13.310	11.155	11.254
H	13.607	9.769	10.584
O	7.057	16.495	11.113
H	7.569	17.277	11.395
H	7.636	15.744	11.335
O	8.364	4.187	8.942
H	8.121	4.144	7.996
H	9.264	4.560	8.966
O	5.747	6.499	12.066
H	4.899	6.972	12.130
H	6.299	7.023	11.459
O	8.661	14.447	5.375
H	9.163	13.749	4.917
H	9.194	15.263	5.347
O	4.815	15.179	10.055
H	4.865	15.279	9.088
H	5.554	15.675	10.439
O	12.952	8.364	13.241
H	13.057	9.074	12.582
H	12.361	7.696	12.845
O	12.999	12.848	14.168
H	13.289	13.091	13.271
H	12.187	12.324	14.005
O	4.037	11.666	4.761
H	3.917	10.738	4.492
H	3.243	11.892	5.278
O	5.452	14.006	5.077
H	4.790	13.346	4.801
H	5.886	14.354	4.280
O	13.293	5.991	10.214
H	13.966	5.499	10.710
H	12.615	6.206	10.883
O	9.321	12.770	15.554
H	9.828	12.200	14.943
H	9.938	13.480	15.786
O	2.847	11.491	10.815
H	2.618	12.441	10.872
H	2.117	11.039	10.357
O	13.300	13.053	11.462
H	12.637	13.753	11.309
H	14.151	13.395	11.129
O	13.036	7.714	4.963
H	12.937	8.274	4.177
H	13.989	7.603	5.127
O	9.218	12.123	4.050
H	8.308	11.880	4.296
H	9.789	11.534	4.576
O	7.640	4.315	6.322
H	6.667	4.233	6.367
H	7.812	5.217	6.003
O	7.210	13.949	14.375
H	8.000	13.618	14.838
H	6.473	13.499	14.816
O	4.901	15.499	7.322
H	3.940	15.419	7.208
H	5.274	14.969	6.598
O	15.985	9.677	5.821
H	15.907	8.716	5.702
H	16.173	9.808	6.764
O	5.867	4.450	10.166
H	6.743	4.385	9.737
H	5.948	5.128	10.861
O	1.852	8.398	7.562
H	2.686	8.388	8.067
H	1.700	7.462	7.330
O	7.458	10.670	16.049
H	7.854	9.782	16.040
H	8.202	11.282	15.913
O	7.023	16.947	8.355
H	6.977	16.793	9.314
H	6.271	16.474	7.954
O	3.729	5.382	8.842
H	4.409	4.898	9.351
H	3.919	6.326	9.000
O	7.205	14.716	3.076
H	7.703	14.509	3.889
H	7.699	15.478	2.726
O	3.725	6.358	4.777
H	4.487	6.109	4.226
H	3.676	7.325	4.699
O	11.897	5.623	6.229
H	11.804	4.724	5.845
H	12.218	6.195	5.510
O	3.234	10.488	13.291
H	3.026	11.202	13.923
H	3.107	10.893	12.413
O	8.479	3.862	12.506
H	8.499	3.930	13.474
H	9.241	3.296	12.259
O	8.605	8.105	15.553
H	9.344	7.568	15.902
H	7.926	7.464	15.277
O	12.083	16.942	10.106

H	12.443	16.662	9.242
H	11.467	17.670	9.930
O	7.954	10.034	1.619
H	8.691	9.907	0.987
H	7.679	10.959	1.531
O	13.955	11.073	4.651
H	14.750	10.550	4.871
H	13.447	10.511	4.044
O	11.423	14.618	15.538
H	11.942	15.051	16.218
H	12.037	14.035	15.050
O	0.899	9.851	9.703
H	0.727	9.170	10.369
H	1.222	9.372	8.919
O	12.896	15.949	7.767
H	12.901	16.197	6.828
H	13.761	15.510	7.927
O	6.149	6.071	3.530
H	6.241	6.712	2.803
H	6.737	6.381	4.248
O	5.252	9.436	14.753
H	4.514	9.958	14.384
H	5.845	10.029	15.242
O	15.945	10.147	8.572
H	15.237	9.558	8.894
H	16.341	10.573	9.347
O	3.568	9.058	4.117
H	2.629	9.190	4.335
H	3.642	8.892	3.159
O	14.282	5.589	7.597
H	13.448	5.437	7.117
H	14.035	5.690	8.527
O	5.589	16.026	13.466
H	6.199	15.375	13.849
H	5.984	16.311	12.627
O	12.697	9.346	2.724
H	12.084	8.650	2.429
H	12.664	10.014	2.022
O	1.651	12.192	6.038
H	1.116	12.163	6.858
H	1.396	11.376	5.583
O	10.740	7.500	1.939
H	10.174	7.806	2.672
H	10.258	6.765	1.537
O	15.111	14.560	8.138
H	15.425	14.376	9.039
H	14.977	13.695	7.713
O	4.944	4.535	6.494
H	4.516	5.135	5.861
H	4.566	4.728	7.366
O	11.287	4.865	14.464
H	11.846	4.161	14.096
H	10.409	4.484	14.635
O	0.731	7.587	11.349
H	0.000	7.377	11.932
H	0.830	6.825	10.743
O	2.623	14.142	11.185

H	2.652	14.439	12.108
H	3.429	14.508	10.765
O	9.028	18.157	11.962
H	9.589	17.611	12.538
H	9.570	18.448	11.214
O	6.843	6.220	14.614
H	6.561	6.246	13.679
H	6.034	6.425	15.124
O	10.052	16.800	5.333
H	11.017	16.685	5.376
H	9.782	17.367	6.074
O	0.080	12.030	8.168
H	0.183	12.886	8.626
H	0.244	11.325	8.814
O	10.982	7.025	16.213
H	11.157	6.247	15.657
H	11.814	7.524	16.262
O	8.638	4.273	15.197
H	7.997	5.000	15.072
H	8.239	3.658	15.815
O	11.515	14.674	2.561
H	11.843	14.243	3.365
H	11.044	13.985	2.064
O	13.107	16.350	12.558
H	12.903	16.634	11.649
H	12.246	16.313	13.003
O	1.088	5.534	9.654
H	0.699	5.451	8.776
H	2.037	5.369	9.522
O	15.436	7.265	13.044
H	14.516	7.524	13.230
H	15.907	8.108	12.965
O	13.098	8.878	15.851
H	13.114	8.617	14.909
H	13.728	9.607	15.914
O	1.122	9.597	5.247
H	1.361	9.211	6.114
H	0.207	9.360	5.092
O	15.874	10.042	12.897
H	15.044	10.085	12.380
H	15.640	10.382	13.775
O	5.595	12.630	16.206
H	5.558	12.978	17.099
H	6.218	11.882	16.227
O	8.926	18.420	7.290
H	8.411	19.052	6.785
H	8.266	17.804	7.677
O	11.717	2.374	8.077
H	11.012	1.865	8.509
H	11.569	3.289	8.361
O	15.162	4.859	11.896
H	15.298	5.697	12.387
H	16.013	4.635	11.514
O	10.083	12.602	1.545
H	9.218	12.774	1.145
H	9.872	12.372	2.470
O	6.686	7.653	1.336

H	7.107	8.525	1.458
H	7.390	7.060	1.029
O	14.608	12.064	7.108
H	15.187	11.434	7.574
H	14.653	11.831	6.165
O	10.660	2.424	12.038
H	11.598	2.648	12.073
H	10.449	2.098	11.140
O	11.685	3.134	5.418
H	10.811	2.898	5.058
H	11.769	2.677	6.276
O	9.548	1.744	9.644
H	9.051	2.553	9.424
H	8.880	1.062	9.841
O	14.788	11.108	15.247
H	15.303	11.591	15.895
H	14.143	11.742	14.877
O	15.602	6.965	5.685
H	15.217	6.498	6.454
H	16.018	6.291	5.146
O	10.440	16.165	13.425
H	10.662	15.750	14.270
H	9.867	15.533	12.962
O	12.208	11.194	0.711
H	11.514	11.807	1.020
H	12.733	11.670	0.066
O	6.338	2.250	11.762
H	5.950	2.904	11.156
H	7.109	2.701	12.142
O	15.679	15.508	12.795
H	14.739	15.778	12.763
H	16.185	16.317	12.868
O	9.294	16.393	2.678
H	10.084	15.877	2.444
H	9.476	16.755	3.559
O	0.833	14.461	9.150
H	1.398	14.397	9.940
H	1.407	14.716	8.412
O	1.901	5.790	6.800
H	2.374	5.880	5.955
H	2.564	5.464	7.431
O	2.263	14.830	6.715
H	2.131	13.897	6.465
H	1.634	15.340	6.202
O	4.014	8.356	1.512
H	4.957	8.134	1.421
H	3.564	7.971	0.759
O	7.357	12.784	1.164
H	7.233	13.435	1.882
H	6.818	13.088	0.431
O	10.605	9.977	15.592
H	9.985	9.241	15.713
H	11.485	9.604	15.780
O	17.130	11.322	10.877
H	16.711	10.843	11.618
H	18.073	11.170	10.947
O	7.379	0.223	10.271

H	6.710	0.000	9.625
H	6.971	0.849	10.900
O	8.767	5.802	0.842
H	8.943	5.378	0.000
H	8.430	5.108	1.439
O	15.794	13.783	10.695
H	15.914	14.372	11.464
H	16.356	13.002	10.816
O	10.135	9.453	0.108
H	10.849	10.101	0.216
H	10.401	8.671	0.623
O	7.510	3.991	2.432
H	6.980	4.643	2.930
H	6.978	3.195	2.376
O	3.054	15.147	13.724
H	2.525	15.891	14.015
H	3.970	15.479	13.629
O	10.307	18.977	9.584
H	10.468	19.920	9.545
H	9.807	18.740	8.783
O	3.137	12.651	14.902
H	3.974	12.686	15.389
H	3.027	13.514	14.469
O	9.082	2.835	4.531
H	8.603	3.235	5.279
H	8.741	3.255	3.733
O	13.182	3.300	13.080
H	13.643	2.545	13.449
H	13.846	3.849	12.633
O	4.529	7.004	15.737
H	3.674	6.771	15.375
H	4.717	7.918	15.455
O	12.743	16.203	5.054
H	13.102	16.569	4.244
H	12.688	15.239	4.912

450

w150 optmized with ChargeNN

O	11.458	10.624	11.662
H	10.990	9.783	11.506
H	10.767	11.304	11.787
O	9.640	12.605	11.897
H	9.759	12.993	11.009
H	8.719	12.282	11.951
O	9.702	11.277	8.280
H	9.919	12.094	8.771
H	9.228	11.568	7.482
O	9.976	8.310	11.231
H	9.193	8.749	10.853
H	10.472	7.967	10.463
O	7.990	9.846	10.051
H	8.460	10.323	9.349
H	7.715	10.510	10.712
O	10.734	8.738	8.610
H	11.704	8.817	8.663
H	10.386	9.638	8.482
O	10.211	13.633	9.476
H	9.669	14.444	9.475
H	11.107	13.937	9.242
O	13.471	12.099	10.616
H	13.881	12.486	11.410
H	12.742	11.540	10.950
O	7.197	11.519	12.033
H	7.344	11.022	12.863
H	6.254	11.756	12.041
O	13.408	9.129	8.678
H	13.500	10.067	8.427
H	13.755	8.616	7.926
O	11.715	11.280	6.190
H	12.472	11.461	6.775
H	10.917	11.533	6.690
O	13.760	11.700	7.957
H	14.706	11.538	7.786
H	13.680	11.964	8.896
O	7.562	10.066	14.256
H	7.032	10.209	15.062
H	8.485	9.955	14.560
O	12.081	10.607	14.335
H	11.959	10.628	13.370
H	11.308	10.123	14.679
O	9.996	8.948	14.929
H	10.035	8.176	14.342
H	9.932	8.608	15.841
O	10.195	14.741	13.431
H	9.872	14.652	14.344
H	9.984	13.901	12.978
O	12.653	15.071	12.270
H	11.772	14.941	12.667
H	12.761	16.031	12.158
O	10.217	6.692	13.349
H	10.194	7.095	12.455
H	10.297	5.723	13.239
O	12.884	14.420	9.309
H	13.078	13.691	9.924
H	13.154	15.235	9.771
O	5.793	8.233	10.353
H	5.947	8.285	11.317
H	6.538	8.726	9.969
O	8.957	12.318	5.948
H	8.369	11.930	5.276
H	9.198	13.199	5.599
O	14.677	8.947	11.073
H	14.098	9.043	10.294
H	14.516	8.050	11.415
O	9.939	7.126	6.640
H	10.156	7.597	7.469
H	10.348	7.669	5.948
O	10.673	9.125	4.903
H	9.984	9.587	4.394
H	11.101	9.816	5.441
O	6.184	8.175	13.007
H	5.291	8.149	13.401
H	6.663	8.914	13.435
O	12.754	7.016	14.258
H	11.816	7.043	14.001
H	12.816	6.372	14.990
O	13.843	14.561	6.813
H	14.447	13.843	6.564
H	13.598	14.371	7.740
O	11.814	6.192	10.401
H	12.174	6.185	9.496
H	12.570	6.106	10.997
O	15.177	10.541	13.208
H	14.904	11.469	13.123
H	14.846	10.069	12.422
O	8.488	16.581	12.491
H	8.822	17.491	12.600
H	9.181	15.979	12.834
O	9.444	4.735	10.297
H	9.192	4.749	9.351
H	10.299	5.194	10.358
O	7.668	6.176	14.059
H	7.087	6.915	13.797
H	8.578	6.446	13.834
O	11.031	14.989	6.941
H	11.959	14.719	7.008
H	11.043	15.962	6.876
O	6.024	15.431	11.889
H	6.078	15.360	10.923
H	6.866	15.805	12.193
O	14.324	8.967	15.348
H	14.385	9.684	14.692
H	13.687	8.332	14.976
O	13.334	12.718	15.354
H	13.784	13.029	14.553
H	12.853	11.911	15.070
O	5.508	11.198	7.450
H	5.280	10.739	6.627
H	4.701	11.629	7.781
O	6.979	13.390	7.541
H	6.417	12.590	7.612
H	7.818	13.076	7.164
O	14.224	6.431	12.097
H	15.022	6.125	12.565
H	13.617	6.674	12.823
O	9.276	11.880	15.908
H	10.021	11.711	16.514
H	9.269	12.835	15.736
O	4.459	11.708	12.270
H	3.972	12.426	11.832
H	3.942	10.903	12.095
O	14.474	13.222	12.887
H	13.860	13.964	12.704
H	15.378	13.585	12.812
O	14.434	7.911	6.474
H	14.376	8.165	5.539
H	15.383	7.974	6.689
O	11.465	11.926	3.541
H	10.520	11.722	3.429
H	11.639	11.762	4.486
O	8.632	4.898	7.722
H	7.669	5.061	7.779

H	9.044	5.690	7.336
O	9.013	14.657	15.912
H	9.774	14.639	16.516
H	8.206	14.490	16.441
O	6.205	15.406	9.130
H	5.265	15.542	8.910
H	6.470	14.624	8.608
O	16.326	10.806	7.597
H	16.556	9.959	7.173
H	16.596	10.673	8.522
O	7.212	4.723	11.754
H	8.021	4.738	11.200
H	7.384	5.303	12.520
O	3.296	8.552	9.311
H	4.256	8.491	9.475
H	2.979	7.631	9.235
O	7.447	11.665	17.867
H	7.874	11.081	18.516
H	8.094	11.718	17.135
O	8.762	15.889	9.878
H	8.698	16.137	10.818
H	7.849	15.718	9.585
O	4.972	5.658	10.652
H	5.731	5.153	11.003
H	5.272	6.587	10.636
O	9.241	14.803	4.974
H	9.791	14.931	5.772
H	9.686	15.381	4.327
O	5.318	7.582	6.368
H	6.227	7.505	6.011
H	5.053	8.490	6.161
O	13.125	6.012	7.986
H	12.968	5.154	7.543
H	13.448	6.623	7.305
O	4.136	11.827	15.014
H	4.118	12.759	15.301
H	4.296	11.847	14.053
O	10.312	3.981	13.705
H	10.470	3.919	14.661
H	11.117	3.626	13.275
O	9.793	7.772	17.362
H	10.679	7.529	17.707
H	9.309	6.945	17.210
O	12.862	17.821	12.077
H	13.449	17.573	11.339
H	12.148	18.320	11.634
O	9.030	10.718	3.309
H	8.974	10.290	2.439
H	8.144	11.073	3.507
O	15.819	12.789	5.881
H	16.081	12.054	6.467
H	15.640	12.387	5.018
O	11.511	13.959	16.918
H	11.342	13.272	17.586
H	12.147	13.532	16.309
O	2.781	9.552	11.777
H	3.044	8.970	12.512
H	3.044	9.104	10.949
O	14.145	16.835	9.937
H	14.060	17.161	9.026
H	15.104	16.797	10.127
O	7.696	7.277	5.206
H	7.999	7.503	4.310
H	8.495	7.204	5.762
O	5.809	10.216	16.345
H	5.166	10.827	15.937
H	6.315	10.733	17.001
O	17.024	9.796	10.003
H	16.195	9.658	10.499
H	17.304	8.898	9.745
O	4.901	9.999	4.994
H	3.935	10.138	5.013
H	5.089	9.437	4.216
O	15.249	5.834	9.611
H	14.496	5.989	9.008
H	14.886	5.910	10.509
O	7.501	16.881	15.070
H	7.931	16.120	15.495
H	7.802	16.874	14.147
O	14.800	8.744	3.864
H	13.940	8.463	3.499
H	14.878	9.701	3.725
O	3.418	12.756	8.372
H	3.314	12.903	9.330
H	2.776	12.060	8.152
O	12.306	7.907	3.160
H	11.744	8.303	3.855
H	11.867	7.074	2.929
O	16.767	16.694	10.589
H	16.887	16.749	11.550
H	17.174	15.862	10.294
O	5.954	5.451	8.010
H	5.661	6.231	7.510
H	5.583	5.518	8.903
O	12.939	5.178	16.224
H	13.602	4.627	15.778
H	12.130	4.646	16.315
O	3.649	8.004	13.903
H	2.994	8.440	14.480
H	3.402	7.064	13.771
O	3.735	16.618	12.674
H	3.954	17.004	13.536
H	4.563	16.188	12.378
O	11.420	18.195	14.442
H	11.724	17.570	15.118
H	12.015	18.095	13.679
O	8.316	5.530	16.644
H	7.998	5.710	15.737
H	7.561	5.709	17.231
O	11.220	17.679	6.564
H	12.119	17.776	6.913
H	10.602	17.995	7.251
O	2.605	13.256	10.979
H	2.366	14.186	10.775
H	1.801	12.716	10.892
O	12.271	7.195	18.168
H	12.718	6.577	17.570
H	12.706	8.063	18.088
O	10.481	3.915	16.461
H	9.731	4.529	16.581
H	10.259	3.121	16.948
O	13.550	15.489	4.202
H	13.615	15.153	5.110
H	13.596	14.723	3.611
O	14.476	17.107	14.251
H	13.921	17.414	13.514
H	13.875	16.824	14.958
O	3.707	5.395	13.232
H	4.241	4.774	13.759
H	4.074	5.385	12.333
O	16.761	7.759	15.294
H	15.889	8.149	15.498
H	17.228	8.427	14.765
O	13.171	9.793	17.680
H	13.469	9.532	16.788
H	13.786	10.501	17.938
O	1.832	10.530	8.208
H	2.380	9.784	8.524
H	1.289	10.778	8.972
O	17.687	9.517	13.393
H	16.773	9.851	13.310
H	18.238	10.321	13.394
O	6.653	14.185	17.116
H	5.778	14.207	16.696
H	6.764	13.304	17.515
O	9.435	18.175	8.548
H	8.561	18.359	8.164
H	9.325	17.308	8.977
O	11.896	1.924	8.627
H	10.995	1.882	9.005
H	12.494	1.935	9.389
O	16.452	5.794	13.542
H	16.523	6.440	14.280
H	17.142	6.073	12.921
O	13.499	13.269	2.490
H	13.241	13.623	1.637
H	12.699	12.851	2.865
O	8.459	7.520	2.584
H	8.764	8.192	1.955
H	9.140	6.829	2.597
O	17.479	14.565	7.177
H	17.743	15.248	6.561
H	16.894	13.955	6.696
O	12.765	3.215	12.857
H	13.530	3.423	13.413
H	13.099	2.795	12.046
O	12.890	3.606	6.838
H	12.360	3.662	6.031
H	12.433	2.963	7.425
O	9.666	1.940	10.109
H	9.522	2.882	10.293

H	9.477	1.442	10.921
O	14.853	11.963	17.536
H	15.735	11.781	17.211
H	14.340	12.326	16.792
O	17.082	8.293	6.955
H	17.420	7.739	7.682
H	17.340	7.885	6.108
O	12.591	16.434	16.258
H	12.209	15.548	16.390
H	12.790	16.769	17.134
O	15.325	11.502	3.443
H	14.626	12.095	3.114
H	16.060	11.585	2.833
O	7.803	2.802	13.516
H	7.505	3.357	12.769
H	8.670	3.177	13.751
O	16.965	16.656	13.361
H	16.059	16.817	13.696
H	17.539	17.249	13.847
O	10.969	16.616	3.960
H	11.878	16.280	3.895
H	10.956	17.179	4.752
O	2.218	15.839	10.467
H	2.699	16.298	11.180
H	2.709	15.979	9.641
O	2.643	5.915	9.292
H	2.440	5.154	8.750
H	3.409	5.674	9.845
O	3.707	15.523	8.085
H	3.558	14.570	7.952
H	3.967	15.908	7.232
O	5.585	8.231	2.973
H	6.507	8.013	2.772
H	5.169	7.407	3.277
O	6.798	11.852	4.224
H	6.512	12.768	4.388
H	6.087	11.261	4.533
O	10.992	11.474	18.035
H	10.334	10.963	18.540
H	11.749	10.871	17.906
O	18.374	7.238	12.124
H	19.265	7.072	12.437
H	18.099	8.081	12.541
O	8.785	0.507	12.350
H	8.050	0.000	12.005
H	8.418	1.258	12.846
O	10.666	5.697	2.727
H	10.598	5.132	1.956
H	10.824	5.093	3.477
O	17.014	14.058	12.554
H	17.089	14.958	12.917
H	17.230	14.124	11.605
O	11.977	10.067	1.531
H	11.881	10.770	2.194
H	12.211	9.257	2.020
O	6.952	4.792	4.195
H	7.274	5.641	4.540
H	5.995	4.898	4.128
O	4.824	17.139	15.198
H	4.634	17.684	15.962
H	5.799	17.072	15.141
O	10.827	19.060	10.785
H	10.154	19.188	11.472
H	10.363	18.843	9.958
O	4.164	14.479	15.649
H	3.401	14.417	15.045
H	4.501	15.373	15.487
O	8.890	3.338	5.475
H	8.805	3.702	6.376
H	8.137	3.699	4.972
O	14.857	3.864	14.659
H	15.443	3.191	15.008
H	15.426	4.546	14.259
O	6.181	6.174	18.231
H	5.522	5.500	18.397
H	5.784	6.767	17.556
O	13.943	17.298	7.195
H	14.481	17.734	6.531
H	13.945	16.349	6.976
O	8.997	9.762	19.043
H	8.389	9.289	19.633
H	9.288	9.082	18.406
O	2.321	9.789	15.455
H	2.089	9.807	16.383
H	2.895	10.561	15.296
O	5.066	7.646	16.341
H	4.578	7.601	15.505
H	5.362	8.575	16.419
O	2.236	10.377	5.489
H	1.389	10.511	5.062
H	2.074	10.414	6.449
O	6.542	14.503	5.101
H	7.463	14.754	4.896
H	6.577	14.162	6.014
O	17.713	14.171	9.891
H	18.220	13.348	9.969
H	17.598	14.317	8.935
O	17.590	7.176	9.453
H	16.782	6.626	9.480
H	17.982	7.099	10.338
O	13.921	2.005	10.623
H	14.429	2.475	9.934
H	14.199	1.088	10.591
O	5.296	3.512	14.579
H	4.919	2.646	14.727
H	6.203	3.371	14.258
O	9.402	9.490	0.877
H	9.162	9.793	0.000
H	10.360	9.650	0.976
O	11.265	3.669	4.476
H	10.384	3.582	4.911
H	11.479	2.783	4.180
O	18.934	11.703	10.205
H	18.238	11.021	10.156
H	19.720	11.327	9.807
O	17.110	7.322	4.413
H	17.057	6.451	4.019
H	16.338	7.824	4.098
O	0.836	11.136	10.755
H	1.474	10.539	11.195
H	0.000	11.022	11.210
O	6.815	17.721	7.681
H	6.619	17.009	8.310
H	6.234	17.562	6.927
O	2.421	14.396	13.582
H	2.738	15.269	13.280
H	2.438	13.841	12.791
O	18.804	11.968	12.981
H	18.987	11.912	12.031
H	18.174	12.701	13.079
O	4.431	6.048	4.299
H	3.556	5.682	4.414
H	4.667	6.495	5.136
O	4.956	16.599	5.815
H	5.501	15.872	5.459
H	4.700	17.147	5.072
O	9.285	19.075	13.098
H	10.040	18.843	13.678
H	8.815	19.786	13.535
O	15.145	3.262	8.542
H	14.451	3.330	7.862
H	15.318	4.163	8.859
O	7.286	7.885	20.158
H	6.897	7.332	19.461
H	7.580	7.301	20.856

900

w300 optmized with ChargeNN

O	4.682	21.947	11.885
H	4.728	21.159	11.322
H	3.880	21.817	12.422
O	17.665	16.612	22.842
H	17.156	15.955	23.349
H	18.540	16.203	22.718
O	7.484	21.562	8.823
H	7.317	22.302	9.443
H	7.544	20.770	9.384
O	21.148	12.785	6.195
H	20.920	11.848	6.061
H	21.736	12.852	6.959
O	14.822	23.175	16.642
H	15.717	23.531	16.807
H	14.943	22.218	16.530
O	15.727	15.422	19.859
H	16.003	16.300	20.180
H	15.205	15.583	19.050
O	8.827	19.039	16.667
H	9.296	18.236	16.960
H	8.960	19.078	15.703
O	21.015	20.876	10.441
H	21.567	21.605	10.154
H	20.925	21.000	11.417

O	6.882	23.259	10.809
H	7.424	23.553	11.555
H	6.050	22.915	11.181
O	20.510	10.459	9.508
H	21.267	11.037	9.697
H	20.357	9.979	10.345
O	12.815	17.846	18.519
H	13.080	18.048	19.433
H	13.401	17.125	18.217
O	13.638	16.288	2.920
H	13.196	16.544	3.753
H	13.368	15.368	2.724
O	8.381	17.176	8.804
H	7.981	16.285	8.789
H	9.020	17.216	8.068
O	13.107	13.638	2.682
H	13.619	12.815	2.694
H	12.395	13.503	3.328
O	2.718	21.212	13.605
H	3.312	20.556	14.014
H	2.152	21.553	14.296
O	3.436	16.294	19.575
H	3.270	17.235	19.392
H	3.408	15.912	18.679
O	20.275	11.045	20.565
H	20.885	11.465	19.939
H	19.694	11.743	20.911
O	17.436	18.963	5.544
H	17.678	18.828	4.601
H	16.574	18.509	5.640
O	9.999	6.864	6.993
H	10.199	7.814	7.072
H	9.029	6.808	6.934
O	5.836	15.209	16.225
H	6.027	16.137	16.461
H	6.000	15.080	15.274
O	12.603	17.586	5.167
H	13.475	17.603	5.602
H	12.479	18.462	4.762
O	16.122	17.299	8.613
H	16.951	16.870	8.890
H	15.507	17.255	9.368
O	12.170	3.603	7.781
H	12.615	2.874	8.248
H	11.399	3.852	8.319
O	14.317	10.385	5.897
H	13.534	10.740	6.355
H	14.177	9.424	5.778
O	9.589	26.432	13.256
H	9.388	27.154	12.660
H	10.559	26.389	13.325
O	15.531	17.940	1.811
H	14.875	17.349	2.216
H	15.265	18.069	0.900
O	8.263	9.155	20.819
H	7.815	8.290	20.896
H	9.208	8.950	20.924

O	9.815	16.601	13.180
H	10.035	16.611	12.228
H	9.393	15.738	13.355
O	16.056	12.752	19.855
H	15.970	12.628	18.892
H	15.811	13.682	20.007
O	16.278	25.380	9.621
H	16.356	24.810	8.840
H	16.888	25.001	10.275
O	20.223	5.946	9.072
H	20.301	6.792	8.597
H	20.834	5.967	9.828
O	14.435	17.552	10.717
H	14.441	16.940	11.471
H	14.765	18.411	11.049
O	6.460	15.358	22.989
H	7.426	15.420	23.095
H	6.252	14.472	22.656
O	14.658	8.563	21.604
H	15.573	8.621	21.925
H	14.561	9.313	20.983
O	15.820	1.108	11.096
H	16.395	0.377	10.870
H	15.414	0.905	11.959
O	22.028	15.683	9.667
H	21.555	15.135	10.323
H	21.585	16.546	9.677
O	7.052	19.132	22.224
H	6.984	19.002	23.170
H	6.424	18.508	21.816
O	2.068	9.627	10.944
H	2.298	9.050	11.694
H	1.888	10.503	11.315
O	5.316	8.531	9.668
H	4.451	8.531	9.207
H	5.293	7.733	10.226
O	7.621	16.870	19.490
H	6.875	16.955	20.119
H	7.894	15.937	19.524
O	17.617	10.925	17.598
H	16.697	11.225	17.495
H	18.153	11.714	17.398
O	10.166	13.053	24.183
H	9.404	12.553	24.476
H	10.359	12.778	23.267
O	7.102	3.495	15.311
H	7.841	3.491	15.950
H	6.819	2.582	15.226
O	5.822	16.967	6.569
H	6.053	17.854	6.250
H	5.174	17.084	7.286
O	12.806	2.649	13.038
H	12.154	3.119	13.585
H	12.956	3.208	12.255
O	5.152	11.067	18.034
H	4.233	11.348	18.195
H	5.682	11.888	18.012

O	19.124	5.210	12.618
H	18.533	4.937	13.340
H	18.561	5.285	11.831
O	13.261	13.661	8.461
H	14.044	14.002	8.928
H	12.486	14.084	8.869
O	2.619	12.050	18.328
H	2.881	12.703	19.010
H	1.876	11.567	18.691
O	12.869	14.308	14.305
H	12.222	13.735	13.852
H	12.427	15.162	14.478
O	12.487	5.839	6.387
H	11.613	6.227	6.573
H	12.404	4.900	6.647
O	11.873	11.886	10.710
H	12.838	11.959	10.870
H	11.649	12.644	10.145
O	16.438	14.402	23.997
H	16.679	13.481	23.797
H	16.043	14.380	24.889
O	2.937	8.662	8.531
H	2.472	8.973	9.331
H	2.726	9.296	7.817
O	12.863	13.528	16.892
H	12.855	13.759	15.941
H	12.252	12.771	16.980
O	9.403	16.924	2.663
H	10.254	17.344	2.441
H	8.821	17.081	1.917
O	11.038	12.688	13.175
H	10.187	13.163	13.225
H	11.192	12.436	12.244
O	7.551	7.867	4.533
H	7.456	7.597	5.470
H	7.499	7.058	4.023
O	11.198	20.525	14.811
H	11.731	20.315	15.600
H	11.748	21.124	14.275
O	4.538	19.650	10.219
H	3.767	19.066	10.302
H	4.514	20.037	9.321
O	14.268	5.678	8.496
H	13.611	5.706	7.772
H	13.929	5.022	9.130
O	11.106	21.960	10.159
H	10.997	22.637	10.850
H	10.508	21.245	10.443
O	8.919	13.043	16.225
H	8.183	13.134	16.863
H	9.650	12.599	16.689
O	11.301	17.380	21.913
H	12.100	17.861	21.635
H	10.557	17.872	21.519
O	2.906	10.242	6.333
H	3.521	10.016	5.620
H	3.061	11.171	6.569

O	9.241	15.556	23.203
H	9.689	14.812	23.633
H	9.914	16.180	22.905
O	4.489	12.288	13.200
H	4.608	11.739	13.998
H	4.812	11.763	12.444
O	3.500	13.636	20.328
H	3.433	14.595	20.169
H	4.277	13.491	20.890
O	14.497	3.431	17.289
H	13.621	3.055	17.066
H	14.643	4.191	16.694
O	10.026	14.669	7.226
H	9.131	14.609	6.848
H	10.322	15.584	7.057
O	18.329	6.877	18.721
H	17.889	6.174	19.229
H	18.335	7.682	19.268
O	6.477	10.343	7.796
H	5.925	9.697	8.267
H	7.359	10.289	8.205
O	1.967	12.280	12.157
H	2.803	12.225	12.657
H	1.468	12.994	12.580
O	18.674	21.353	14.584
H	18.466	21.145	15.509
H	18.268	22.222	14.405
O	8.084	7.927	9.548
H	8.348	8.854	9.388
H	7.217	7.961	9.990
O	18.779	16.666	9.002
H	18.590	15.761	9.321
H	18.992	16.604	8.054
O	9.385	9.069	14.954
H	8.690	9.751	14.886
H	10.226	9.542	14.818
O	23.833	17.787	15.783
H	23.125	17.651	16.426
H	23.981	16.958	15.298
O	10.389	9.609	6.922
H	9.879	9.850	6.127
H	11.137	10.230	6.959
O	24.208	16.074	11.403
H	23.551	15.747	10.771
H	24.104	17.040	11.394
O	9.225	18.692	20.704
H	8.772	18.012	20.168
H	8.536	18.991	21.330
O	8.682	5.785	18.221
H	7.743	5.954	18.020
H	9.148	6.606	17.982
O	8.512	21.283	6.379
H	8.468	22.170	6.016
H	8.251	21.367	7.318
O	16.842	23.219	7.893
H	16.942	22.881	6.988
H	17.575	22.837	8.407
O	7.540	14.836	6.130
H	6.956	15.614	6.168
H	6.974	14.059	6.290
O	21.952	18.200	17.874
H	22.473	18.979	18.083
H	21.847	17.694	18.696
O	16.446	5.195	6.737
H	17.091	4.559	7.092
H	15.669	5.185	7.317
O	15.083	14.201	26.350
H	14.937	14.916	26.970
H	14.210	13.939	26.007
O	5.084	9.340	4.647
H	5.501	10.213	4.752
H	5.819	8.743	4.458
O	13.736	18.499	20.997
H	14.645	18.155	21.035
H	13.827	19.461	20.873
O	16.167	9.458	13.520
H	16.911	9.359	14.141
H	15.403	9.655	14.100
O	18.610	13.334	16.816
H	17.789	13.555	16.336
H	19.287	13.875	16.373
O	19.300	15.996	2.603
H	19.434	15.486	3.420
H	19.114	15.364	1.907
O	8.999	10.203	4.631
H	9.720	10.292	3.988
H	8.562	9.344	4.484
O	18.331	12.888	21.299
H	18.051	12.561	22.171
H	17.508	12.815	20.779
O	3.440	12.841	7.273
H	3.312	13.704	6.841
H	3.271	12.935	8.229
O	11.296	21.054	6.081
H	11.748	20.868	6.925
H	10.342	21.015	6.249
O	3.250	15.596	16.927
H	3.014	16.423	16.467
H	4.179	15.410	16.696
O	8.670	23.020	17.747
H	8.808	22.177	18.218
H	7.729	23.058	17.499
O	4.625	7.444	6.689
H	4.576	8.104	5.977
H	3.947	7.703	7.336
O	2.735	17.860	15.465
H	3.433	18.467	15.158
H	2.359	17.480	14.655
O	13.963	7.799	5.196
H	13.760	7.793	4.244
H	13.466	7.062	5.601
O	10.525	17.023	17.351
H	11.289	17.409	17.818
H	10.408	16.145	17.753
O	15.216	17.519	6.059
H	15.395	17.374	7.006
H	15.391	16.667	5.609
O	4.923	16.481	12.539
H	5.448	17.292	12.666
H	4.002	16.687	12.783
O	10.904	14.226	9.658
H	10.785	15.097	10.084
H	10.532	14.334	8.754
O	17.407	19.641	8.204
H	17.523	19.561	7.242
H	17.032	18.785	8.480
O	24.477	11.240	12.255
H	25.410	11.081	12.108
H	24.182	10.557	12.891
O	12.005	2.535	16.784
H	11.825	2.914	15.907
H	11.150	2.588	17.232
O	18.632	16.829	19.215
H	18.740	15.863	19.156
H	17.831	17.007	19.738
O	12.194	7.526	17.655
H	11.266	7.825	17.639
H	12.355	7.172	18.546
O	15.842	16.489	15.579
H	15.255	17.007	14.998
H	16.744	16.831	15.426
O	17.639	8.544	11.408
H	17.100	8.012	10.799
H	17.103	8.726	12.200
O	19.297	18.575	17.223
H	18.984	17.973	17.922
H	20.268	18.540	17.271
O	16.102	12.269	13.221
H	15.464	12.326	12.484
H	16.146	11.319	13.429
O	21.083	17.124	20.319
H	20.995	17.774	21.020
H	20.202	17.025	19.911
O	20.985	14.041	11.526
H	20.088	13.737	11.756
H	21.479	13.247	11.247
O	17.748	20.831	17.205
H	18.302	20.031	17.233
H	16.874	20.568	16.852
O	5.547	17.092	21.166
H	4.742	16.809	20.697
H	5.723	16.430	21.867
O	13.532	8.402	2.517
H	12.728	8.521	2.004
H	13.888	9.292	2.688
O	23.884	12.864	14.420
H	23.791	12.248	15.168
H	24.101	12.316	13.646
O	11.612	10.566	14.718
H	11.431	11.365	14.184
H	11.792	9.863	14.058

O	11.394	9.076	0.774
H	11.892	9.352	0.000
H	10.951	8.255	0.552
O	7.299	6.626	20.507
H	6.539	6.655	19.908
H	7.987	6.181	19.986
O	17.829	5.405	10.143
H	17.209	6.108	9.883
H	18.698	5.668	9.779
O	7.189	19.162	10.232
H	6.224	19.245	10.112
H	7.492	18.417	9.677
O	20.209	9.192	11.862
H	19.341	8.780	11.698
H	20.041	9.885	12.527
O	6.586	18.650	12.875
H	6.731	18.934	11.955
H	7.442	18.756	13.323
O	18.107	9.405	15.415
H	17.980	9.972	16.200
H	18.788	8.745	15.680
O	18.542	21.731	9.532
H	19.403	21.468	9.891
H	18.154	20.922	9.154
O	3.926	5.361	12.955
H	4.547	5.628	12.248
H	3.270	4.798	12.542
O	20.725	18.181	10.101
H	20.617	19.149	10.071
H	19.932	17.797	9.680
O	15.278	14.514	10.078
H	16.245	14.451	9.964
H	15.124	14.990	10.916
O	2.978	8.043	13.068
H	3.112	7.091	12.970
H	3.690	8.344	13.652
O	16.988	4.297	14.125
H	16.594	4.112	13.253
H	16.937	3.460	14.622
O	8.140	11.889	21.171
H	8.105	10.936	20.972
H	9.064	12.070	21.417
O	19.480	14.174	19.190
H	19.007	13.695	19.894
H	19.111	13.865	18.339
O	18.348	17.267	15.036
H	18.692	17.877	15.714
H	18.732	17.540	14.182
O	8.846	14.171	13.774
H	8.897	13.879	14.704
H	7.891	14.210	13.578
O	12.952	6.856	20.236
H	13.656	7.370	20.670
H	13.315	5.966	20.099
O	11.286	10.698	3.072
H	11.160	10.281	2.210
H	12.253	10.742	3.192

O	14.067	21.174	20.428
H	13.353	21.441	19.822
H	14.146	21.879	21.074
O	19.581	11.000	13.729
H	20.181	11.584	14.230
H	19.061	10.493	14.377
O	5.313	10.979	10.941
H	6.246	10.940	11.217
H	5.125	10.110	10.542
O	7.781	10.795	12.070
H	7.819	11.079	13.003
H	8.217	11.487	11.541
O	20.972	21.242	13.015
H	21.640	20.672	13.445
H	20.216	21.298	13.622
O	6.119	22.943	16.751
H	5.292	23.262	17.117
H	6.051	23.073	15.781
O	14.030	11.015	3.254
H	14.948	11.101	2.913
H	14.159	10.881	4.214
O	11.049	11.617	17.219
H	11.228	10.989	16.496
H	11.172	11.150	18.066
O	16.883	4.819	19.825
H	15.914	4.793	19.834
H	17.175	4.019	19.383
O	17.476	23.658	16.882
H	17.749	22.751	17.085
H	17.624	23.806	15.933
O	16.440	13.861	15.362
H	16.183	14.796	15.263
H	16.257	13.409	14.515
O	14.375	20.356	13.684
H	14.849	20.281	12.831
H	13.721	21.065	13.570
O	14.100	17.707	13.963
H	13.213	17.512	14.321
H	14.167	18.677	13.883
O	17.259	9.094	22.290
H	17.890	8.552	22.766
H	17.629	9.224	21.395
O	12.547	24.020	8.963
H	12.735	23.799	8.050
H	11.990	23.309	9.324
O	21.576	15.836	13.467
H	21.454	15.200	12.738
H	22.535	15.865	13.652
O	16.688	7.416	5.252
H	16.723	6.514	5.629
H	15.739	7.593	5.119
O	9.261	6.977	13.265
H	9.811	7.155	12.479
H	9.488	7.654	13.930
O	3.469	18.608	18.135
H	4.399	18.422	17.927
H	2.983	18.478	17.307

O	19.337	17.075	6.339
H	20.144	17.492	6.030
H	18.611	17.641	6.021
O	6.600	11.613	4.955
H	7.485	11.230	4.834
H	6.491	11.760	5.912
O	2.234	16.776	12.939
H	2.066	17.120	12.047
H	1.764	15.928	13.037
O	14.090	11.701	23.769
H	13.614	12.401	24.250
H	13.456	10.998	23.571
O	23.724	9.696	14.294
H	23.319	9.995	15.124
H	23.166	8.984	13.930
O	19.758	17.689	12.682
H	20.450	17.108	13.046
H	20.032	17.843	11.767
O	7.241	7.017	7.067
H	6.266	7.026	6.972
H	7.401	7.327	7.976
O	5.012	13.724	4.010
H	5.561	14.031	3.269
H	5.439	12.912	4.330
O	22.524	12.047	10.462
H	22.884	12.506	9.684
H	23.266	11.766	11.024
O	10.083	5.004	9.084
H	10.366	5.624	9.775
H	9.938	5.549	8.293
O	12.428	20.002	3.891
H	11.930	20.429	4.617
H	13.215	20.533	3.759
O	23.016	13.572	8.225
H	22.632	14.376	8.623
H	23.800	13.850	7.751
O	7.695	11.134	14.738
H	6.757	11.046	15.000
H	8.062	11.875	15.254
O	12.581	6.264	15.166
H	12.346	6.747	15.974
H	13.508	5.984	15.292
O	16.037	7.395	9.519
H	15.366	6.797	9.126
H	16.416	7.887	8.762
O	5.307	5.601	15.367
H	4.694	5.440	14.628
H	5.957	4.880	15.334
O	16.856	11.187	6.534
H	15.919	10.977	6.364
H	17.111	11.792	5.813
O	22.108	6.711	15.544
H	22.139	7.044	14.631
H	22.981	6.823	15.920
O	12.788	24.386	15.412
H	12.113	24.374	16.110
H	13.605	24.067	15.848

O	10.954	8.541	21.182
H	11.497	7.823	20.812
H	11.406	8.820	21.997
O	6.127	6.688	17.894
H	5.718	6.376	17.072
H	6.296	7.642	17.802
O	19.697	7.629	16.433
H	19.447	7.212	17.274
H	20.524	7.210	16.134
O	12.020	14.877	21.061
H	11.679	15.729	21.389
H	12.916	14.801	21.442
O	7.019	14.150	2.131
H	7.153	13.318	1.678
H	7.778	14.280	2.725
O	17.989	14.268	9.906
H	18.141	13.500	9.318
H	18.212	13.945	10.797
O	10.386	16.706	10.574
H	9.595	16.932	10.048
H	11.102	17.255	10.200
O	6.403	20.231	16.549
H	6.328	21.187	16.694
H	7.352	20.014	16.538
O	17.347	8.674	7.496
H	17.142	8.146	6.694
H	17.153	9.596	7.243
O	12.631	20.608	8.434
H	13.563	20.853	8.573
H	12.107	21.108	9.091
O	11.027	4.021	14.688
H	10.313	4.100	14.026
H	11.528	4.860	14.650
O	2.282	17.916	10.307
H	1.533	18.377	9.924
H	2.699	17.410	9.584
O	8.393	14.222	19.615
H	8.362	13.491	20.254
H	9.313	14.289	19.303
O	21.546	5.577	11.544
H	20.681	5.467	11.994
H	21.988	4.730	11.613
O	24.169	15.596	14.098
H	24.427	15.739	13.168
H	24.243	14.643	14.270
O	12.763	16.190	23.986
H	13.481	15.913	23.391
H	12.165	16.733	23.454
O	5.090	20.674	7.771
H	5.412	20.194	6.995
H	5.851	21.182	8.103
O	9.579	8.263	17.494
H	9.501	8.458	16.540
H	8.794	8.680	17.893
O	11.237	13.008	4.615
H	11.077	12.203	4.092
H	10.455	13.577	4.476
O	16.690	20.309	19.796
H	17.075	20.598	18.950
H	15.830	20.746	19.889
O	12.642	9.278	23.254
H	12.692	8.785	24.074
H	13.409	9.005	22.714
O	9.487	20.124	11.342
H	9.405	19.861	12.271
H	8.692	19.794	10.894
O	22.857	19.484	13.862
H	23.234	18.983	14.607
H	23.124	19.025	13.054
O	19.927	15.151	22.180
H	19.495	14.352	21.841
H	20.408	15.570	21.453
O	15.267	20.429	16.227
H	15.116	20.392	15.264
H	14.402	20.222	16.620
O	16.463	17.788	20.761
H	16.894	17.580	21.619
H	16.672	18.716	20.535
O	5.318	8.220	14.607
H	5.299	7.316	14.965
H	5.926	8.202	13.845
O	5.867	23.183	14.098
H	5.382	22.737	13.381
H	6.692	23.513	13.701
O	8.311	24.019	13.125
H	8.561	24.946	13.293
H	8.669	23.496	13.871
O	12.101	5.707	12.510
H	12.336	6.193	13.320
H	11.513	6.287	11.999
O	18.420	12.008	8.577
H	19.196	11.438	8.720
H	17.971	11.713	7.761
O	10.830	23.783	12.096
H	9.900	23.829	12.390
H	11.222	24.647	12.320
O	10.733	12.543	21.576
H	11.116	11.888	20.965
H	11.186	13.383	21.375
O	21.171	12.839	14.856
H	20.801	13.704	15.102
H	22.069	13.017	14.520
O	13.906	25.335	10.956
H	14.742	25.574	10.507
H	13.359	24.928	10.256
O	16.198	2.044	15.457
H	15.647	1.543	14.834
H	15.619	2.382	16.161
O	16.772	11.711	23.246
H	16.995	10.799	23.016
H	15.809	11.700	23.411
O	12.258	18.044	9.194
H	13.080	17.813	9.663
H	12.355	18.982	8.938
O	15.496	19.958	11.320
H	15.470	20.521	10.528
H	16.425	19.922	11.634
O	13.381	14.356	5.833
H	12.585	13.971	5.436
H	13.362	14.138	6.780
O	5.099	10.775	15.340
H	5.015	10.872	16.306
H	5.063	9.817	15.151
O	14.422	8.485	11.523
H	14.951	8.065	10.825
H	15.048	8.876	12.154
O	15.135	5.531	15.665
H	15.612	6.260	16.100
H	15.783	5.121	15.059
O	17.446	23.707	14.075
H	17.880	23.916	13.231
H	16.511	23.637	13.828
O	6.123	12.906	7.380
H	5.147	12.957	7.317
H	6.295	11.996	7.700
O	16.177	3.778	11.579
H	16.785	4.236	10.973
H	16.161	2.836	11.326
O	9.389	22.590	15.123
H	9.971	21.814	15.060
H	9.157	22.711	16.058
O	5.726	6.260	11.141
H	6.365	5.582	10.837
H	6.227	6.865	11.720
O	17.950	18.339	3.059
H	18.493	17.557	2.843
H	17.117	18.237	2.563
O	12.694	20.078	17.034
H	12.700	19.232	17.518
H	12.452	20.759	17.688
O	11.905	21.894	18.899
H	11.763	22.746	18.451
H	11.025	21.479	18.975
O	10.396	7.207	10.777
H	9.536	7.404	10.355
H	11.011	7.863	10.397
O	10.925	14.626	18.618
H	11.395	14.746	19.465
H	11.521	14.118	18.044
O	9.360	3.549	16.839
H	9.210	4.312	17.428
H	9.914	3.856	16.100
O	9.113	14.550	3.917
H	9.294	15.434	3.538
H	8.563	14.694	4.710
O	7.022	8.207	12.478
H	7.831	7.774	12.815
H	7.260	9.142	12.327
O	18.259	9.302	3.926
H	18.884	9.638	4.583
H	17.737	8.595	4.344

O	14.473	14.990	22.216
H	15.129	14.697	22.875
H	14.984	15.188	21.414
O	12.676	13.717	25.102
H	11.765	13.415	24.972
H	12.700	14.617	24.722
O	20.038	8.422	7.801
H	19.065	8.427	7.715
H	20.257	9.106	8.460
O	16.601	10.823	10.220
H	17.228	11.252	9.611
H	17.054	10.050	10.596
O	10.654	17.278	7.125
H	11.253	17.569	7.838
H	11.177	17.368	6.309
O	7.171	9.259	18.299
H	7.423	9.314	19.239
H	6.459	9.911	18.167
O	12.674	22.453	13.548
H	12.721	23.123	14.263
H	12.021	22.812	12.916
O	20.076	15.222	15.563
H	20.709	15.526	14.886
H	19.344	15.857	15.483
O	17.326	13.138	4.665
H	16.677	13.865	4.653
H	18.197	13.568	4.719
O	6.718	13.230	17.769
H	6.303	13.954	17.258
H	7.177	13.645	18.522
O	4.809	15.177	10.162
H	4.822	15.571	11.054
H	4.325	14.328	10.218
O	8.736	12.771	10.432
H	9.594	13.201	10.279
H	8.085	13.392	10.056
O	5.702	12.904	21.925
H	6.525	12.542	21.557
H	5.342	12.240	22.515
O	3.608	16.656	8.285
H	3.365	16.120	7.511
H	4.067	16.059	8.909
O	3.518	12.839	10.014
H	4.179	12.152	10.226
H	2.791	12.674	10.644
O	16.623	11.211	2.815
H	16.928	11.946	3.374
H	17.196	10.455	3.046
O	16.522	7.570	16.817
H	17.153	7.236	17.484
H	17.060	8.117	16.219
O	17.877	19.733	12.511
H	18.150	20.344	13.219
H	18.504	18.993	12.539
O	3.782	15.209	5.905
H	4.540	15.814	5.933
H	3.978	14.639	5.137

O	11.984	8.748	12.853
H	11.694	8.925	11.939
H	12.929	8.559	12.742
O	22.690	11.266	16.447
H	21.951	11.691	15.978
H	22.588	11.536	17.369
O	6.173	14.379	13.616
H	5.747	15.132	13.167
H	5.565	13.622	13.505
O	18.284	9.395	19.821
H	19.110	9.854	20.069
H	17.948	9.887	19.051
O	4.654	19.547	14.585
H	5.203	19.838	15.336
H	5.285	19.258	13.904
O	14.662	15.506	12.562
H	14.677	16.285	13.146
H	14.059	14.894	13.015
O	12.075	9.213	10.189
H	11.963	10.179	10.236
H	13.006	9.028	10.400
O	8.737	4.334	13.226
H	8.840	5.305	13.245
H	8.046	4.103	13.868
O	14.120	4.401	19.753
H	14.209	4.094	18.828
H	13.791	3.647	20.246
O	13.606	4.267	10.858
H	13.085	4.891	11.399
H	14.514	4.254	11.216
O	15.352	12.429	17.302
H	15.758	12.937	16.572
H	14.406	12.632	17.230
O	12.117	11.679	6.905
H	11.766	12.222	6.176
H	12.517	12.302	7.537
O	9.134	19.066	13.927
H	9.916	19.603	14.162
H	9.463	18.173	13.698
O	14.291	15.784	17.650
H	14.863	16.035	16.897
H	13.797	14.994	17.372
O	22.209	7.788	13.023
H	22.167	6.979	12.487
H	21.494	8.344	12.660
O	14.423	9.076	17.643
H	15.177	8.501	17.413
H	13.632	8.504	17.630
O	1.826	13.508	16.009
H	2.033	12.800	16.636
H	2.292	14.291	16.362
O	6.527	19.492	5.682
H	6.381	19.644	4.747
H	7.340	19.973	5.918
O	15.262	21.220	8.867
H	15.913	20.585	8.523
H	15.625	22.091	8.623

O	18.544	13.049	12.292
H	17.622	12.965	12.591
H	18.980	12.284	12.707
O	14.341	10.585	19.889
H	14.483	10.160	19.021
H	14.936	11.356	19.930
O	8.871	10.404	9.012
H	9.567	10.283	8.339
H	9.019	11.246	9.474
O	12.257	25.895	13.097
H	12.942	25.844	12.402
H	12.632	25.521	13.914
O	14.268	10.207	15.177
H	13.325	10.381	15.024
H	14.344	9.880	16.092
O	17.624	23.661	11.357
H	16.714	23.416	11.602
H	17.972	22.949	10.796
O	18.437	3.740	8.013
H	18.177	4.130	8.859
H	19.279	4.150	7.792
O	7.728	4.534	10.608
H	8.135	4.282	11.452
H	8.452	4.600	9.962
O	14.485	12.141	11.073
H	14.686	12.998	10.651
H	15.149	11.521	10.705
O	11.772	16.706	14.950
H	11.353	16.829	15.821
H	11.043	16.707	14.298
O	6.042	17.644	17.370
H	6.609	17.443	18.137
H	6.314	18.520	17.049
O	15.091	23.464	12.500
H	14.687	24.174	11.962
H	14.348	22.969	12.876
O	7.258	14.681	9.179
H	7.032	14.228	8.343
H	6.404	14.874	9.607
O	13.511	1.888	9.509
H	14.329	1.457	9.772
H	13.439	2.676	10.072
O	9.487	20.629	18.788
H	9.288	20.051	18.028
H	9.387	20.056	19.571
O	23.310	18.663	11.182
H	22.401	18.603	10.846
H	23.650	19.517	10.915
O	15.559	15.234	4.633
H	15.144	15.556	3.815
H	14.824	14.806	5.120
O	0.949	14.401	13.631
H	0.000	14.371	13.765
H	1.340	14.081	14.473
O	11.654	10.747	19.744
H	12.627	10.718	19.821
H	11.327	9.926	20.154

O	21.834	12.637	18.861
H	21.088	13.260	18.833
H	22.530	13.069	19.361
O	17.198	21.760	5.502
H	17.431	22.056	4.622
H	17.276	20.793	5.505
O	11.095	24.243	17.671
H	10.182	23.907	17.605
H	11.049	25.063	18.166
O	19.598	14.633	4.969
H	20.207	13.981	5.366
H	19.482	15.348	5.613
O	11.769	18.119	1.948
H	12.450	17.456	2.145
H	11.927	18.864	2.550
O	20.497	10.098	5.656
H	21.093	9.665	5.041
H	20.368	9.486	6.403
O	14.592	0.754	13.535
H	13.931	1.462	13.374
H	14.096	0.000	13.856

References

(1) Liang, Q.; Yang, J. Third-Order Many-Body Expansion of OSV-MP2 Wave Function for Low-Order Scaling Analytical Gradient Computation. *J. Chem. Theory Comput.* **2021**, *17*, 6841–6860.

(2) Feng, H.; Zheng, Y.; Wang, Y.; Li, S.; Li, W. Efficient Computational Strategies of the Cluster-in-Molecule Local Correlation Approach for Interaction Energies of Large Host–Guest Systems. *J. Chem. Theory Comput.* **2025**, *21*, 2998–3009.

(3) Sheng, X.; Kazemi, M.; Żądło Dobrowolska, A.; Kroutil, W.; Himo, F. Mechanism of biocatalytic Friedel–Crafts acylation by acyltransferase from Pseudomonas protegens. *ACS Catal.* **2019**, *10*, 570–577.
